# Supplementary figures and images for: Fine-grained, nonlinear registration of live cell movies reveals spatiotemporal organization of diffuse molecular processes
Source: PLoS Comput Biol. 2022 Dec 30;18(12):e1009667. doi: 10.1371/journal.pcbi.1009667 (PMC9870159; doi:10.1371/journal.pcbi.1009667)

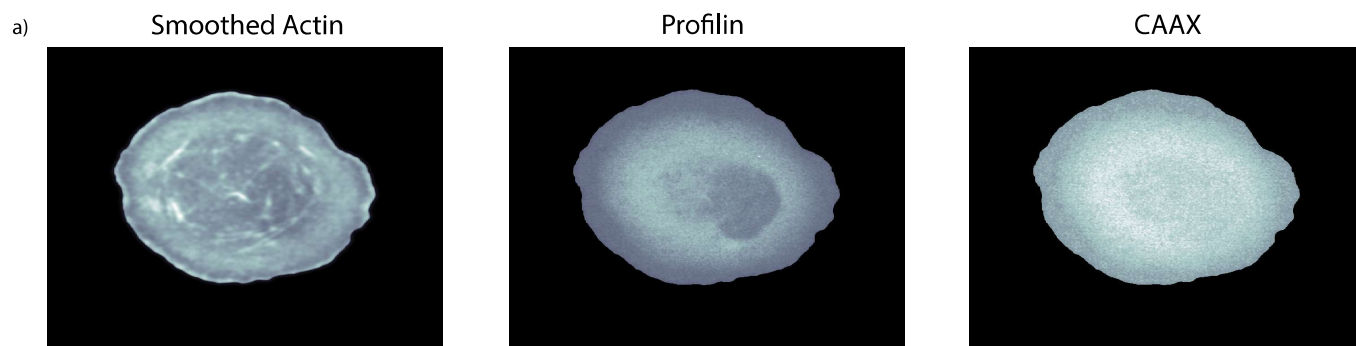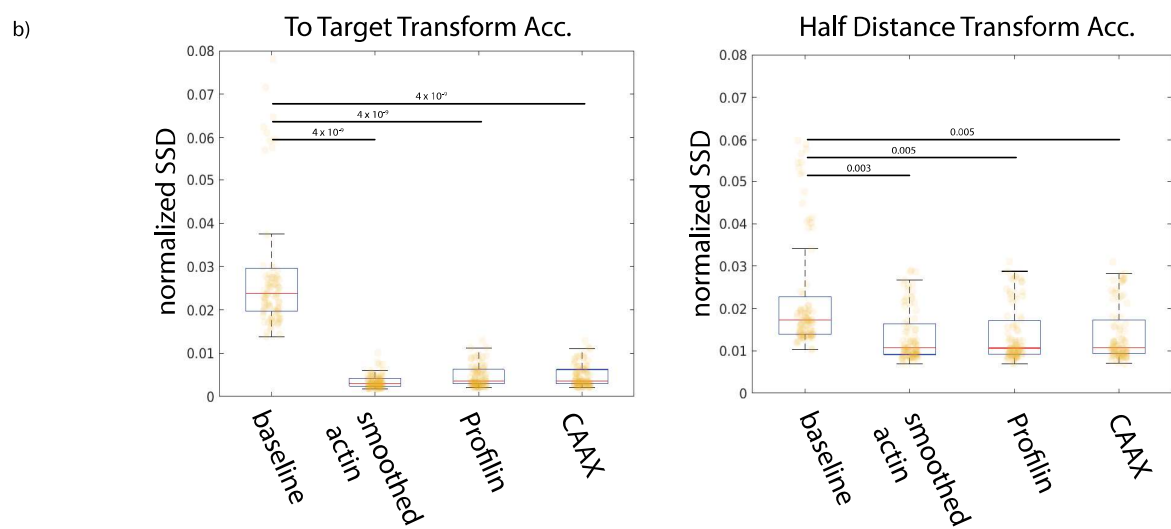

Supplement: S1 Fig — a) Reference frame images of the three location fiducials, lowpass-filtered Actin, Profilin, and CAAX in the same U2OS cell displayed in Fig 1b) To-target transformation and half-distance transformation accuracies (sum of squared distance (SSD) between target and remapped images) computed for the full mNG-Actin signal of interest (see Fig 1) using different location fiducials as indicated. The SSD between untransformed moving and target frames is computed as a baseline. Box plots illustrate 25th, 50th, and 75th percentile of n = 91 moving/target frame pairs pooled from m = 4 movies. Whiskers indicate the 5th and 95th percentile. P-values are calculated by one-way ANOVA testing. (PDF) [file pcbi.1009667.s001.pdf]

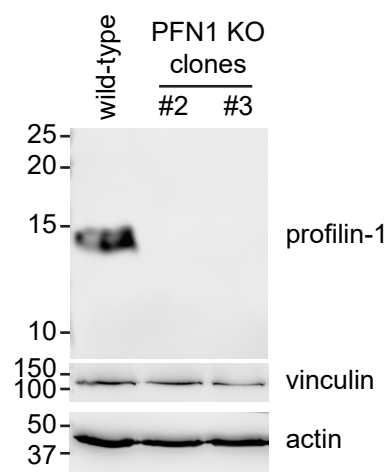

Supplement: S2 Fig — Profilin knockout was verified using western blotting using mouse monoclonal anti-Profilin-1 antibodies. Vinculin and Actin provided as loading control. See materials and methods for antibody source. (PDF) [file pcbi.1009667.s002.pdf]
