# Supplementary material for: Fine-grained, nonlinear registration of live cell movies reveals spatiotemporal organization of diffuse molecular processes
Source: PLoS Comput Biol. 2022 Dec 30;18(12):e1009667. doi: 10.1371/journal.pcbi.1009667 (PMC9870159; doi:10.1371/journal.pcbi.1009667)
Supplement: Attachment — Submitted filename: rebuttal.final.pdf [file pcbi.1009667.s010.pdf]

Reviewer's Responses to Questions

Comments to the Authors:
Please note here if the review is uploaded as an attachment.
Reviewer #1: This article addresses an important yet often underappreciated problem of cell mapping for fluorescent images in cases where no simple ground truth position marker is available, and where the focus is on tracking of diffuse protein dynamics. While pixel scale dynamics is often notable by eye in these movies, quantitative analysis and comparison across experiments is difficult due to changes in cell shape. The method introduced by the authors represents an important advance to the field. However, the constraints and limitations of the new method are not described in the manuscript, and the improvements over other approaches appear exaggerated.

We appreciate this input. We have added a paragraph to the Discussion sections on the limitations, of which there are numerous. We also apologize if we come across as overselling the approach. In our revision we have attempted to avoid such phrases, but we are certainly open to the reviewer's specific input on this issue. Our intention with this paper is to provide the community with a tool for cell registration and we try to illustrate the enabling value of the tool with applications of time series analyses in morphologically active cells that were, to our knowledge, not previously considered (local time series coherence and entropy analyses to capture spatial organization in fairly diffuse cytoplasmic image signals). In terms of the registration, what's new, to our knowledge, is the regularization of optical flows via the gradient of the distance transform to the cell boundary and the explicit enforcement of diffeomorphism via sorting. We have tried to better and more neutrally summarize these contributions in the revised Discussion.

While the authors describe the assumptions for the pipeline in lines 117 onward, they state assumptions that are never completely fulfilled in particular the assumption that the cell sits flat in 2D. There are two specific concerns. First, as described, the images appear to be traditional fluorescence images of cells that are fully in the field of view (that is never stated or explained, but should be). Thus changes in fluorescence intensity can also indicate changes in the local thickness of the cell. Does this mean that the cell registration projects changes in thickness as lateral displacements of the cell? How accurately does a 2D optical flow model capture the 3D flow inside the cell that is being captured? Would this approach also be applicable to confocal images?

The reviewer is, of course, correct. Our choice of a 2D analysis entails several limitations that are not specific to the proposed registration pipeline, but implicit to 2D imaging. To be quite honest, on a fundamental level, the concern arises as to how relevant any experiments are with a cell culture on flat (and stiff) surfaces. Such surfaces impose significant, non-physiological constraints on cytoskeleton organization, molecular distribution, and molecular activation. Nonetheless, there are still many valid cell biological experiments undertaken that rely on such culturing conditions, and our work seeks to elevate the flexibility and opportunities for local time sampling dependent analyses in such settings. More immediately relevant to the 3D

concern raised by the reviewer, on such surfaces cells tend to spread to unnaturally flat morphologies. From an image analytical viewpoint, this means that most of the action is restricted to the lateral dimension. With the exception of the nuclear region and of a scenario of vertical ruffling the image pattern exploited for the fine-grained registration thus undergoes lateral deformations that are accounted for by the proposed algorithm. Moreover, subcellular regions with violation of this assumption are effectively imputed. We expect the alignment accuracy to deteriorate with larger regions of weakly or undefined image patterns, but the algorithm is fairly robust against a complete collapse. This claim is supported by Fig. 3d, where we show that the registration driven by diffuse or structured image patterns serving as location fiducials are of comparable quality. The reason for this performance lies in our introduction of optical flow regularization via the distance transform gradient and explicit enforcement of diffeomorphism via sorting.

To answer the reviewer's question regarding confocal microscopy, as outlined in the Methods, all data used in this study has been acquired by spinning disk confocal microscopy with a depth of field of ~800nm – 1 micron. Like in the vast majority of experiments using this type of imaging modality, the microscopist focused on an imaging plane that maximizes the footprint of the cells to ensure that adherent protrusions in lateral directions are fully captured. Inherently, this yields information loss in more central parts of the cell that tend to be out-of-focus. To some extent this could be mitigated by acquisition of full 3D stacks, either by confocal or high-resolution light-sheet imaging, followed by projection, such as maximum intensity projection, into a 2D image. Nonetheless, the projection step will also cause loss of information about 3D rearrangements of the cytoplasm. Which of the two strategies, single-plane confocal or projection, causes bigger artifacts for the proposed registration will also depend on the cell type and/or labelling. This is certainly a factor users (of any 2D cell image analysis) must stay alert to. In the same vein, there is no fundamental roadblock to applying the proposed registration to time lapse sequences acquired by TIRF or regular epifluorescence imaging. We have added some explanation to the effects of the imaging modality to the same paragraph on limitations in the Discussion.

The second concern is that such a remapping may not be suitable for proteins that are bound to the surface directly or indirectly - the laboratory reference frame would appear to be more suitable for analysis of such proteins.

The reviewer is correct in this point, if s/he means proteins that are bound to the cell's ventral surface *and* the substrate. For any other protein, bound for example to the dorsal surface, the proposed registration provides a mechanism to compensate for whole cell morphological changes and/or intra-membrane flow. In the revised version of the manuscript, we now discuss the exceptional scenario of a protein cohort stabilized to the lab frame of reference as the ultimate limitation for this method – in fact, in this scenario there is no need for registration to perform time series analyses.

Additional comments:
The authors describe their approach as image registration, which is more commonly used to

compensate for fluctuations in the imaging field of view rather than compensating for cell movement and deformation. Since traditional image registration is also an important aspect of any data analysis it will be important for the authors to clearly differentiate their effort on cell registration from image registration work.

We appreciate this point and explicitly introduce the term *cell registration* in the revised manuscript.

The authors describe the prevalent analysis approach to such data as “anecdotal” and “superficial” which is not an accurate representation of the quantitative prior work in this area, including work by the authors. I recommend a more nuanced description of prior work in this area.

We appreciate the reviewer pointing this out. We are glad the reviewer recognized that we included our own work in descriptions under these terms. There was no intention of dismissing the cited previous work but we sought to delineate where the proposed work moves the needle with respect to previous work. We fully agree that this language was not helpful, and we apologize if we unintentionally mischaracterized some of the reviewer’s work. We have paid close attention to avoiding this language.

Since optical flow is the key enabling technique, an expanded discussion with references of the following statement would be important – line 72 “However, because of the aperture problem and image noise, Optical Flow computation is ill-posed and thus requires domain-specific regularization techniques.”

We suspect that the term ‘aperture problem’, introduced in the classic optical flow paper by Horn and Schunck, is the stumbling block in the quoted sentence. Newer literature on optical flow tends not to discuss this central limitation, but focuses on optical flow as an ill-posed inverse problem that requires regularization. We have taken a similar strategy to avoid a lengthy discussion of why optical flow is not defined perpendicular to the local intensity gradient.

Coherence is a 3 x 3 region if I understand the explanation – This will introduce asymmetries. Did the authors verify that the asymmetry of this metric does not lead to artifacts when it is applied along boundaries? In other words, Does coherence depend on the angle of the cell boundary relative to the image axes?

We do not fully understand the reviewer’s concern about asymmetry. However, the reviewer is correct that coherence cannot be computed at the most outer rim of the cell edge, where the 3x3 neighborhood is not fully defined. We place the central element of the 3 x 3 neighborhood only on pixels that are more than one pixel away from any background pixels. This generates 1 pixel-wide band of undefined coherence along the cell edge. We have updated the Methods to more explicitly reflect this.

Line 93 “appreciate” - the scientific meaning of this term is unclear.

We replaced ‘appreciate’ with ‘capture’.

The abstract describes a study of “signaling” as separate from “protein distributions” but the experiments shown only focus on fluorescently labeled proteins. Please clarify

The reviewer is correct. The original purpose for our development has been the analysis of subcellular signaling domains, which are difficult to delineate without stabilization of the cell geometry. As the Reviewer probably knows, we have a long-standing collaboration with Klaus Hahn’s lab using FRET-based biosensors to quantify subcellular signaling dynamics. As we were working through the preparation of this manuscript, we got very excited about the new possibilities of mapping out subcellular patterns in the dynamics of largely diffuse cytoplasmic proteins such as Profilin. To demonstrate the broader range of application we have now added an example characterizing the spatial organization of activation of a GEF and downstream GTPase, probed by two multiplexed FRET-based biosensors (new Fig. 4). Of note this analysis is performed on previously published data (Marston, Vilela et al. Nat. Chem. Bio, 2020) and we limit the presentation here to demonstrating the effects of cell registration on increasing the time-series coherence. Numerous applications are arising from this, which are currently under investigation in our lab. It is too early to present these next steps already here; and doing so would also detract from the core message of cell registration.

Reviewer #2: An interesting article well satisfying criteria for publication in PLOS Comp Biol. It describes an improved method of elastic registration based on optical flow with the help of the motion mask as a location fiducial while maintaining diffeomorphism using sorting-based regularization.

Authors show the efficiency of the algorithm with the help of ‘to-target transform’ and ‘half-distance transform’ applied to a sequence of U2OS cells and convincingly demonstrate its applicability to showing dynamics of Profilin and Actin in these cells.

Thank you very much for this supporting assessment and for the thoughtful comments on some flaws in the original submission. We greatly appreciate the Reviewer’s time and effort that went into this.

I would have the following comments:

In p. 11, lines 201-204 you mentioned panels i-iv, but there are no such panels in Fig. 2.

These labels have been added now.

In the same page, l. 209, you mentioned „2 distant time points“. Please, use a word for the number and I propose to specify this time distance, also l. 214.

We have followed the Reviewer's suggestion and indicate the elapsed time in frames and seconds.

In Figure 4 there should be probably „Vol. Marker mCherry Coherence" as a title for the top-right picture. Possibly, the Intensity and Coherence pictures in the last row were swapped (R88E), since the dispersions in the graph below appear not to correspond to visualized pictures.

The panel heading has been changed. However, the Reviewer's assertion that the two panels for R88E were swapped is incorrect. The Reviewer may not have considered that the Gini coefficient of statistical dispersion normalizes the overall signal. While the coherence level is reduced from wildtype to mutant (see Fig. 5c and 5d, now Fig. 6c and 6d), the spatial heterogeneity of the coherence level remains higher than the one of the fluorescence intensity levels also for the mutant. In fact, this illustrates the power of the proposed approach in detecting spatial structure in largely diffuse signals by exploitation of locally coupled time series.

In p. 38, l. 767: In my opinion, "the band 0 – 3.6 µm" does not correspond to the previously stated "1.2 µm from the cell edge". Please, complete also the description of the vertical axes of the graphs in e).

The discrepancy between figure panels and caption has been resolved. Also we have added more detail to the caption to describe the graphs, as suggested.

I had trouble joining the description both in the text (p. 19) and the legend with Figure 6. It is not clear to me what is the cell front and from the small pictures what exactly happens. All the visualized cells are practicaly the same when printed on paper. I propose to apply here, e.g., zoomed-in insets, arrows in the pictures that would help readers to understand visualized and described processes, though it is true that Movie 8 helps with this.

We have updated this figure (now Fig. 7) with a timeline that guides the viewer through the phases of symmetry breaking. Based on the Reviewer's feedback we noted that our description was partially confusing. Following the drug-induced symmetry breaking protocol we had established in Lomakin et al. Nat. Cell Bio 2015 we started filming cells as they were already 20 minutes into the cytoskeleton rearrangement. The peak of this activity is observed in the first half of the movie, previously referred to as 'before symmetry breaking', while the second half of the movie displays convergence of cell organization to steady state polarization, previously referred to as 'after symmetry breaking'. We clarify now that the 4 min time point is in fact at the peak of rearrangement activity related to symmetry breaking. We have added further overlays to the 21 min time point indicating the newly emerged cell front and rear. The text has been updated accordingly. We hope that this will help the Reviewer and reader better follow the analyzed dynamics.

It appears to me that Movie 1 and Movie 2 – top row are identical.

The Reviewer is correct. This was an oversight during uploading of the supplemental videos. The revised version no longer contains this duplication of data.

Please, correct these small typos:
p. 8, l. 134: structures
p. 10, l. 191: put the reference before the dot
p. 17, l. 344: selected

Thank for catching these. They are all corrected.

Reviewer #3: In this manuscript, the authors present a method for registering cellular images adapting from optical flow-based approaches with an updated regularization component that includes the constrain of cell boundaries location. Overall the proposed methods look quite promising in realigning/transforming the cellular images to a reference image of cell at the reference time points. However, the utility of the proposed method in analyzing and interpreting cell dynamics and biological applications was not very clear and the study of the profillin dynamics by applygin the proposed registered images is very descriptive and lack of stasistical significance.

We thank the Reviewer for the overall supportive assessment. We address the specific points below. However, in responding to these introductory comments, we have taken the concern regarding lacking statistical significance to heart and added both individual cell data points and significance tests to all relevant graphs. Second, we also appreciate the comment on a lack of clarity regarding the biological relevance of the Profilin analysis. For the Reviewer we summarize the key concept: Profilin is a ~15 kDa molecule implicated in the regulation of actin dynamics. Its localization in the cytosol is diffuse and thus it has been very challenging to capture any form of spatiotemporal regulation of, for example, concentration shifts, etc. It should be noted that this is a characteristic of many molecular regulators and thus the described analysis is prototypical for a large class of investigations.
Because of the fine-grained cellular registration we introduce in this manuscript we were able to perform pixel-level time series analyses that exploit time integration to unveil patterns of spatial localization. The two integration measurements we introduce are the coherence, measuring co-fluctuations of diffuse signals in a narrow neighborhood, and the Shannon information entropy, which measures the local temporal 'stability' of a structured signal. We emphasize once again, the computation of these measurements has been enabled by the geometric registration of the entire cell footprint.
We then could show the spatiotemporal coupling of a diffuse regulator (Profilin) and a structured macromolecular target assembly (actin cytoskeleton) (new Fig. 6), how the coupling vanishes with expression of a mutant that blocks regulator-target interaction, and how this coupling varies over time as the cell reorganizes regulator and target during an event like drug-induced cell polarization. We believe that this prototype analysis has the potential of inspiring numerous cell biological studies of similar scope.

Returning to the concern of ‘descriptive experiments’. We agree that the polarization study is descriptive at this point and limited to the presentation of one representative example. We are actively working on numerical methods to track spatiotemporal shifts in subcellular microdomains of distinct regulator-target interactions. With such a framework we will then be able to provide integrated statistical information across cell populations. However, these algorithms are a) involved, deserving their own publication; and b) still need better validation in our lab. As the present manuscript is focused on the cell registration, which is statistically validated, we hope the Reviewer will agree with us that two admittedly anecdotal (but qualitatively reproducible) examples of analyses downstream of the core methods will help rather than distract from providing a showcase of the image analytical opportunities this work generates.

Here are my main concerns:

1) Though the authors show that using their updated method, they can re-map the cell images toward the reference images with great similarity, it is not clear the biological representaion of these transformed images referenced at the target images as the ground truth is difficult to establish. The utility of the proposed method to study system dynamics should be further validated in biological dynamics systems with a known/expected outcome.

The reviewer is correct in the assessment that the key challenge of introducing this algorithm has been the definition of analyses and experiments as surrogates of an absolute ground truth. While we appreciate the request for more experiments (and we have provided more) we would like to point out that our original manuscript was designed to provide this rightfully requested validation.
We started the validation in Fig. 3 by analyzing the remapping accuracies for the to-target and the half-distance transforms for various choices of location fiducial. These analyses provide a metric of internal consistency of the mapping. We have embellished this analysis with an example where we could also use fluorescent CAAX as a fiducial marker (new Suppl. 1).
In Fig. 4 (now new Fig. 5) we then introduced the local coherence as an image filtering approach enabled by the proposed cellular registration method to extract hidden spatial structure in diffuse image signals. We showed that the spatial dispersion (Gini coefficient) of the coherence scores increases for a diffuse but locally organized molecule compared to a volume marker. These analyses provide evidence that the registration is sufficiently accurate and fine-grained to capture patterns of coordinated intensity fluctuations within 3x3 pixel neighborhoods. We have embellished this type of analysis by coherence score measurements for signaling activities visualized by FRET-based biosensors (new Fig. 4).
In Fig. 5 (now new Fig. 6) we showed with the use of a Profilin mutant known to abrogate its interaction with actin that the overall level of Profilin coherence significantly decreases, i.e. the local organization of the diffused signal is diminished, as well as the temporal correlation between Profilin coherence and entropy of the actin intensity. The latter result is one of these biological dynamical systems with known/expected outcome, as requested by the reviewer.

The fact that our analyses do report this outcome validates the entire pipeline from cellular registration to coherence and entropy analysis.

We thus feel our (revised) manuscript provides a critical performance assessment of the pipeline, in absence of a formal ground truth. Clearly, gaining the reader's confidence in the pipeline is the crux of this paper. One could always do more, but we hope that with these additional analyses and statistical evaluations (see below), we are able to convince this reviewer.

2) The authors applied their registration method to study the profilin dynamics where they used the CAAX membrane marker as their fiducial images. However, The author only evaluated the performance of using profilin, smoothed actin, and VASP as fiducial points in figure 3, and the performance of CAAX as fiducial points was not evaluated. Hence, it's unclear how well the registration performs based on CAAX images to study the profilin dynamics.

This is a very valid point. We have added a new Suppl. Fig 1 with experiments akin to Fig. 3 showing the registration performance with a CAAX-based fiducial marker.

3) "..we hypothesized that the same principle of spatial coherence in dynamic behavior could be applied to map out the subcellular organization of protein dynamics…" the spatial coherence appears to be a key characteristic that authors measured to assess the dynamics of profillin but what this characteristic represents is unclear. "…as expected, coherence of a diffuse signal is high across the entire cell footprint….". It's not clear what to expect here. The authors should provide a more detailed explanation of the meaning of spatial coherence. In the method section for coherence measurement, the author mentioned that the coherence is measured using a 3x3 matrix of 9-time series centered on the target pixel and measure correlation coefficient for all 72 =non-identical time-series pairs from 9-time points images. It's not clear how 3x3 and 9-time points is arrived and it seems that there should be only 36 non-identical pairs.

We have expanded the Methods on the coherence analysis, also to more explicitly address the cell boundary problem raised by Reviewer #1.

4) Its general lacking of statistical description/significance in the profillin dynamic study using the proposed algorithm (figure 4 to figure 6). In Figures 4 and 6, it seems there is only one cell is measured in each comparing condition, and hence the statistical significance of the observed phenomenon is unclear.

We have added dots to all the box plots in Fig. 3 – 6, provide explicit definition of sample sizes, and we have added statistical test values. The old Fig. 6, now new Fig. 7 is indeed a single case example of symmetry breaking. The goal of this figure is to showcase the types of analyses that are enabled by cell registration. As discussed above, while the presented example is visually representative for several of these experiments, proper statistical analysis of the relocation of the zones of high Profilin coherence and high Actin entropy during drug-induced symmetry

breaking is beyond the scope of this work. In revising the manuscript text we have made sure to refrain from any biological interpretation/conclusion based on this data.

In Figure 5, it seems there are multiple measurements (Figure 5e), but there is no description of sample size and the number of independent replicates. There is also no statistical test to evaluate the significance of differences. Overall, more cells from biological replicates should be measured to further validate the finding.

See previous response. Sample sizes and statistical significance are now indicated for all comparative analyses in Figs 3 – 6.

5) The figure caption for figure 1D is not clear and does not reflect the statement, “The mapping into a rigid frame of reference permits straightforward sampling of times series”.

We have slightly rephrased the caption text. We would appreciate feedback on whether the new version is clearer.

6) There is no statistical information provided for figure 3D. What do the measurements in the box plot represent?

See above. Sample sizes and statistical significance are now indicated for all comparative analyses in Figs 3 – 6.

7) Figure 4A, the figure title label seems to be wrong

Thank you. This has been corrected, also in response to Reviewer #2.
