# Supplementary material for: Fine-grained, nonlinear registration of live cell movies reveals spatiotemporal organization of diffuse molecular processes
Source: PLoS Comput Biol. 2022 Dec 30;18(12):e1009667. doi: 10.1371/journal.pcbi.1009667 (PMC9870159; doi:10.1371/journal.pcbi.1009667)
Supplement: Attachment — Submitted filename: rebuttal.final.docx [file pcbi.1009667.s011.docx]

**Comments to the Authors:**

**Reviewer #1:**

The revised manuscript addresses all concerns of this reviewer and provides a clear introduction to the valuable new tool developed by the authors. I would like to request the authors review one particular wording choice and correct several minor errors.
The manuscript could be clearer on the nature of the corrections introduced by the authors. The goal is stated in the introduction – highlighting molecular interactions. As I understand, the authors wish to remove advection effects from the motion (which would impact all interacting molecules in the same way) and only retain reactions, active transport e.g. via motors or passive diffusion. It should be clarified that advective subcellular movements can also be of importance.

We thank the reviewer for this comment. S/he is absolutely correct that advective subcellular movements are for many questions central. If this is the case, users can exploit the spatially resolved displacement field for further analysis. We have added explanations to this effect in the Introduction and the Discussion.

The following minor errors should be addressed based on the redlined copy:
In the abstract the wording is incorrect: “in function of”

Triggered by this comment, we have edited the abstract and also the Author Summary to sharpen the message further.

Line 72 there is a word missing “the method enables the registration the cell outline”

Typo corrected.

Line 223 “5 s per frame to 50 s per frame.” This could be misunderstood as a change in the exposure time of the camera.

Thank you for this well-justified comment. The sentence is reformulated to explicitly say that the interval between frames is 10 times increased by the down-sampling.

Line 236 actin appears twice.

Typo corrected.

Line 248 onward – random words are not crossed out.

We appreciate the Reviewer’s pointing out some general text errors. We have taken the opportunity of this revision to correct numerous flawed sentences.

Line 286: there seems to be a mistake in the wording – is the new method still only useful for “the entire cell perimeter” or does the remapping also work for the interior of the cell?

Indeed, we meant to write ‘throughout the entire cell’.

Reviewer #3: This reviewer has no further question.
